# Time to therapeutic effect with daridorexant: Weekly analysis of patient-reported outcomes from a Phase 3 randomized, placebo-controlled study in patients with insomnia disorder

**DOI:** 10.1192/j.eurpsy.2025.506

**Published:** 2025-08-26

**Authors:** T. R. Bakker, O. Briasoulis, A. Olivieri, S. Pain, L. Palagini, D. Kunz, P.-A. Geoffroy

**Affiliations:** 1 Idorsia Pharmaceuticals Ltd., Allschwil, Switzerland; 2 Department of Neuroscience, Section of Psychiatry, University of Pisa, Pisa, Italy; 3 Clinic for Sleep & Chronomedicine, St. Hedwig-Krankenhaus, Große Hamburger Straße 5-11, 10115, Berlin, Germany; 4 GHU Paris - Psychiatry & Neurosciences; 5 Université de Paris, NeuroDiderot, Inserm; 6 Département de Psychiatrie et d’Addictologie, AP- HP, GHU Paris Nord, DMU Neurosciences, Hôpital Bichat - Claude Bernard, Paris, France

## Abstract

**Introduction:**

Daridorexant is a dual orexin receptor antagonist approved for the treatment of chronic insomnia. Phase 3 studies have demonstrated that consistent nightly use of daridorexant 50 mg significantly improved both subjective total sleep time (sTST) and daytime functioning (Insomnia Symptoms and Impacts Questionnaire [IDSIQ] sleepiness domain) at Months 1 and 3 in patients with insomnia disorder versus placebo (Mignot *et al*. Lancet Neurol 2022; 21 125-39).

**Objectives:**

This exploratory analysis evaluated how the effect of daridorexant on both sleep and daytime functioning are perceived by patients with insomnia over time (i.e. week by week), as compared to placebo.

**Methods:**

In this multi-centre, double-blind trial (NCT03545191), adult (18–64y) and elderly (≥65y) patients with insomnia disorder were randomized (1:1:1) to receive daridorexant 25mg, 50mg or placebo every evening for 3 months. Patients completed daily eDiary entries throughout the study, and weekly mean changes from baseline in sTST and IDSIQ-sleepiness domain were used to assess patients’ perceived duration of sleep and daytime functioning, respectively.

**Results:**

Weekly mean changes from baseline of both the sTST (Figure 1) and IDSIQ-sleepiness domain (Figure 2) from Week 1 to Week 12 of treatment improved over time in all treatment groups. At each week, the observed improvements from baseline were numerically larger for daridorexant 25 and 50 mg than placebo, with the 50 mg group demonstrating the greatest response. For sTST, treatment differences versus placebo were seen from Week 1 of treatment (mean increases from baseline for 25 mg, 50 mg vs placebo of +23.83, +32.63 vs 10.07, respectively), and were maintained throughout the study. For IDSIQ-sleepiness domain scores, treatment differences vs placebo were seen from Week 1 (mean reductions from baseline for 25 mg, 50 mg vs placebo of –1.56, –1.90 vs –0.92, respectively), with further separation from placebo until Week 4.

**Image 1:**

**Figure:**
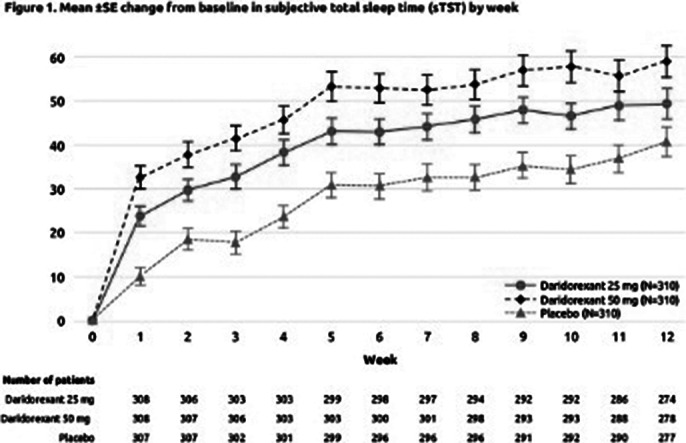

**Image 2:**

**Figure:**
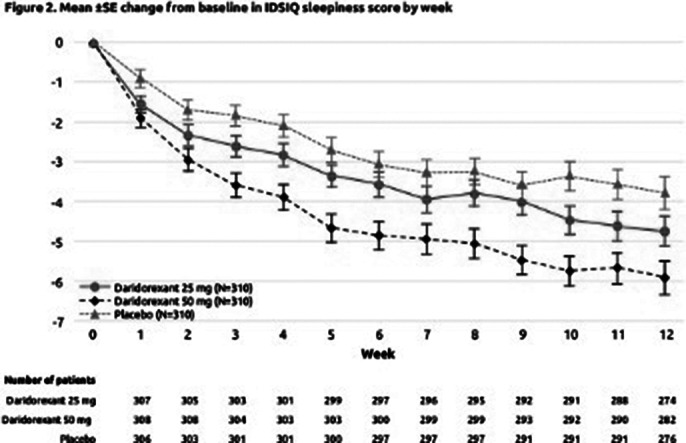

**Conclusions:**

The benefits of daridorexant treatment on total time asleep and daytime functioning start to be perceived from Week 1. With consistent nightly use, efficacy on sleep and daytime functioning continued to build over the course of the 3-month treatment period, with the greatest effect being observed with daridorexant 50 mg.

**Disclosure of Interest:**

T. Bakker Employee of: Idorsia Pharmaceuticals, O. Briasoulis Employee of: Idorsia Pharmaceuticals, A. Olivieri Employee of: Idorsia Pharmaceuticals, S. Pain Employee of: Idorsia Pharmaceuticals, L. Palagini Consultant of: Bruno, Fidia, Idorsia Pharmaceuticals, Pfizer, Sanofi, Pharmanutra, Neopharmed Gentili, D. Kunz Consultant of: Austrian Association of Skiing (ÖSV), Idorsia Pharmaceuticals, Speakers bureau of: AbbVie, Idorsia Pharmaceuticals, German Ministry for Economy (BMWi), Austrian Association of Skiing (ÖSV), P.-A. Geoffroy Consultant of: Apneal, Arrow, Biocodex, Dayvia, Di&Care, Idorsia Pharmaceuticals, Janssen-Cilag, Jazz pharmaceuticals, Myndblue, Mysommeil, Posos, ResilEyes, Withings, Speakers bureau of: Biocodex, Bioprojet, Ibsa, Idorsia Pharmaceuticals, Janssen-Cilag, Isis Medical, Jazz pharmaceuticals, Lundbeck, MySommeil, Withings

